# Lactate, Fructose and Glucose Oxidation Profiles in Sports Drinks and the Effect on Exercise Performance

**DOI:** 10.1371/journal.pone.0000927

**Published:** 2007-09-26

**Authors:** John L. Azevedo, Emily Tietz, Tashena Two-Feathers, Jeff Paull, Kenneth Chapman

**Affiliations:** Exercise Biology Laboratory, Department of Kinesiology, California State University Chico, Chico, California, United States of America; University of Louisville, United States of America

## Abstract

Exogenous carbohydrate oxidation was assessed in 6 male Category 1 and 2 cyclists who consumed CytoMax™ (**C**) or a leading sports drink (**G**) before and during continuous exercise (CE). **C** contained lactate-polymer, fructose, glucose and glucose polymer, while **G** contained fructose and glucose. Peak power output and VO_2_ on a cycle ergometer were 408±13 W and 67.4±3.2 mlO_2_·kg^−1^·min^−1^. Subjects performed 3 bouts of CE with **C**, and 2 with **G** at 62% VO_2_peak for 90 min, followed by high intensity (HI) exercise (86% VO_2_peak) to volitional fatigue. Subjects consumed 250 ml fluid immediately before (−2 min) and every 15 min of cycling. Drinks at −2 and 45 min contained 100 mg of [U-^13^C]-lactate, -glucose or -fructose. Blood, pulmonary gas samples and ^13^CO_2_ excretion were taken prior to fluid ingestion and at 5,10,15,30,45,60,75, and 90 min of CE, at the end of HI, and 15 min of recovery. HI after CE was 25% longer with **C** than **G** (6.5±0.8 vs. 5.2±1.0 min, P<0.05). ^13^CO_2_ from the −2 min lactate tracer was significantly elevated above rest at 5 min of exercise, and peaked at 15 min. ^13^CO_2_ from the −2 min glucose tracer peaked at 45 min for **C** and **G**. ^13^CO_2_ increased rapidly from the 45 min lactate dose, and by 60 min of exercise was 33% greater than glucose in **C** or **G**, and 36% greater than fructose in **G**. ^13^CO_2_ production following tracer fructose ingestion was greater than glucose in the first 45 minutes in **C** and **G**. Cumulative recoveries of tracer during exercise were: 92%±5.3% for lactate in **C** and 25±4.0% for glucose in **C** or **G**. Recoveries for fructose in **C** and **G** were 75±5.9% and 26±6.6%, respectively. Lactate was used more rapidly and to a greater extent than fructose or glucose. CytoMax significantly enhanced HI.

## Introduction

Intense endurance exercise promotes dehydration and depletion of blood glucose, muscle and liver glycogen, and electrolytes. Endurance athletes must satisfy the needs for fluids, energy, and electrolytes for optimal performance. Fluid-energy-electrolyte replacement beverages (i.e., sports drinks) improve endurance because they satisfy these needs, particularly in hot and humid environments [1], [2], [3].

Traditional sports drinks supply energy in the form of sugars (glucose, fructose, sucrose) and glucose polymers [1]. Carbohydrate is the main energy source for prolonged physical activity [4], and of the dietary energy substrates, carbohydrates are most readily digested and absorbed. The drinks also contain electrolytes to replace those lost in sweat. Electrolytes also stimulate thirst, promote solute absorption in the gastrointestinal (GI) tract [1], [2], [3], and buffer endogenous acids [5].

A sports drink containing a 6% (w/v) glucose solution is efficacious for promoting GI emptying and exercise performance. Consumption of 1 liter per hour in 250 ml aliquots delivers 1 g/min of glucose, which enhances fuel availability and provides other benefits [1], [3]. More, recently investigators [6]–[10] have experimented with combinations of hexoses (e.g., 2 glucose/fructose) to raise drink solute content above 6% by taking advantage of specific intestinal transporters that promote solute absorption [11]–[14]. The same investigators have used isotopically labeled solutes to track the oxidation of specific energy substrates in sports drinks. The results support the concept of increasing energy delivery by expanding the metabolite delivery profile of the beverages.

Lactate is a dynamic substrate with great potential as an energy source in sports drinks. To date, however, the efficacy of adding lactate to these drinks has been sparsely assessed [5], [15], [16]. Lactate was once considered a metabolic waste but is now recognized as an important energy substrate in the body. Lactate is the main product of carbohydrate metabolism and can be used as a fuel in working muscle cells shuttled to other tissues such as the heart where lactate is fuel [17], or to the liver were lactate serves as a gluconeogenic precursor [18].

Lactate is transported across cell plasma and mitochondrial membranes by a family of proton-lactate anion-coupled symporter proteins [19], [20], [21], of which MCT1 is the predominant isoform in muscle [22], [23]. Related, but from a different gene family is the sodium-coupled intestinal lactate transporter, sMCT, also known as the slc5a8 [24], [25]. The presence of monocarboxylate (i.e., lactate) transport proteins in the GI tract, erythrocytes, myocytes, cardiocytes, hepatocytes, astrocytes and neurons provide a metabolic rationale for including lactate-containing food additives in sports drinks. PolyLactate™ in CytoMax™ (**C**) might hasten the delivery of substrate during prolonged intense exercise, which may improve sprint performance and delay prevent fatigue after prolonged, hard exercise.

In the present study we evaluated rapidity and extent of use of substrates present in **C** and a popular brand (**G**). The main finding was that lactate was oxidized faster and to a greater extent than fructose or glucose, which are the principle nutrients contained in most sports drinks. Including lactate as a component of sports drinks is logical based on the research showing its role in carbohydrate utilization during exercise (i.e., the Lactate Shuttle) and the present results show that exogenous lactate is a readily available substrate in that it is rapidly transported and oxidized.

## Results

### Subject Characteristics

Participants were fit men (Table 1), with a mean VO_2_max (VO_2_peak) exceeding 5 L O_2_·min^−1^ (67 ml O_2_·kg^−1^·min^−1^). As expected, maximum RER was well over 1.0 during the maximum exercise capacity test. Maximum heart rate varied with age with a mean of approximately 190 bpm (Table 1). All subjects reached at least 400 watts power output during the maximum performance tests; the mean maximum power output was 408 watts. Body weight was 76 kg on average with a range of 65 to 88 kg. Body fat percentages were approximately 14%.

**Table 1:** Subject characteristics.

	VO_2max_	HR_max_	Power_max_	Wt	Body Fat	Age
	(ml·kg^−1^·min^−1^)	(bpm)	(watts)	(kg)	(%)	(yrs)
S1	64.5	178	400	65.5	15.1	33
S2	70.0	180	425	76.0	13.6	25
S3	70.3	203	400	72.0	14.6	24
S4	70.5	191	400	74.0	15.8	26
S5	65.0	195	400	82.2	12.9	26
S6	64.1	178	450	88.0	13.5	44
MEAN±SD	67.4±3.2	187.5±10.4	408±12.9	76.3±7.9	14.3±1.1	29.7±7.7

### Whole-Body Metabolism

**Figure 1:**
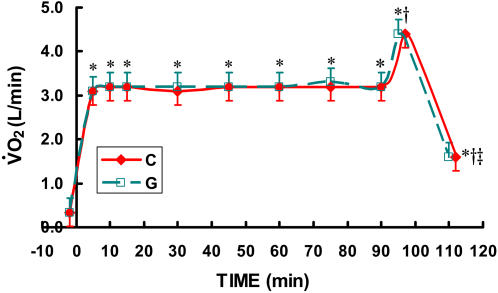
Time-course of VO_2_ (L/min) while exercising at 62% for 90 minutes followed by exercise to exhaustion at 86% of VO_2_max during three CytoMax (C) trials and two G trials. The three C trials and two G trials were randomly ordered. Because no differences existed between trials, the data from the three C trials and two G trials were averaged. Data are means±SE. No difference existed between drinks. * significantly different from rest (p<0.01). † significantly different from steady state exercise (p<0.01). ‡ significantly different from VO_2_ during HI (p<0.05).

Oxygen consumption for all three **C** trials and both **G** trials were combined and presented as “**C**” and “**G**”. Subjects rode at a VO_2_ corresponding to ∼62% of their VO_2_max (3.2 L O_2_·min^−1^, Figure 1) for 90 minutes followed by an effort that elicited ∼85% of their VO_2_max (4.4 L O_2_·min^−1^) until volitional fatigue, typically ∼4–7 minutes. VO_2_ returned rapidly toward resting values after the cessation of exercise (Figure 1).

**Figure 2 pone-0000927-g002:**
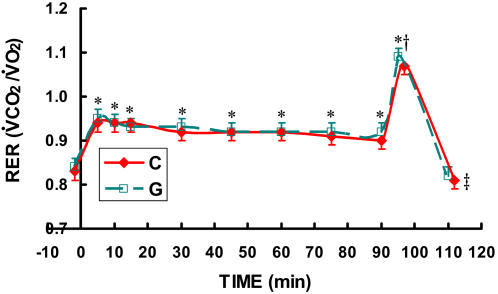
Time-course of respiratory exchange ratio (RER = VCO_2_/VO_2_) while exercising at 62% for 90 minutes followed by exercise to exhaustion at 86% of VO_2_max during three C trials and two G trials. The three C trials and two G trials were randomly ordered. Because no differences existed between trials, the data from the three C trials and two G trials were averaged. Data are means±SE. No difference existed between drinks. * significantly different from rest (p<0.05). † significantly different from steady state exercise with the exception of minutes 5 and 10 (p<0.05). ‡ significantly different from HI (p<0.05).

Compared to rest, respiratory exchange ratios were consistently elevated to about 0.93 for the sustained 90-minute exercise, indicating that the primary fuel utilized during exercise was carbohydrate (Figure 2) in all the **C** and **G** trials. During the HI effort RER exceeded 1.0 in all **C** and **G** trials (Figure 2).

### Blood Metabolites

**Figure 3 pone-0000927-g003:**
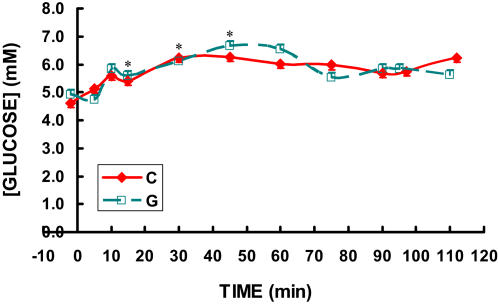
Time-course of blood glucose concentration while exercising at 62% for 90 minutes followed by exercise to exhaustion at 86% of VO_2_max during three C trials and two G trials. The three C trials and two G trials were randomly ordered. Because no differences existed between trials, the data from the three C trials and two G trials were averaged. Data are means±SE. No difference existed between drinks. * significantly different from rest (p<0.05).

**Figure 4:**
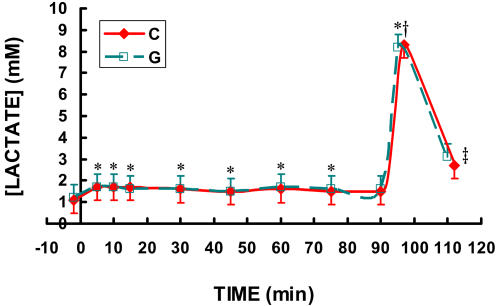
Time-course of blood lactate while exercising at 62% for 90 minutes followed by exercise to exhaustion at 86% of VO_2_max during three C trials and two G trials. The three C trials and two G trials were randomly ordered. Because no differences existed between trials, the data from the three C trials and two G trials were averaged. Data are means±SE. No difference existed between drinks. * significantly different from rest. For beverage G, only 5, 10, & 15 min time points significantly different from rest. For C, all time points indicated are different from rest. † significantly different from steady state exercise (p<0.05). ‡ significantly different from blood lactate during HI (p<0.05).

At rest, blood glucose concentrations in postabsorptive men were slightly below 5 mM, and rose gradually during all **C** and **G** trials to between 6 and 7 mM at 45 min, then gradually declined back toward initial values of approximately 5 mM by 90 min of exercise (Figure 3). Lactate was approximately 1.0 mM at rest and rose slightly to approximately 1.7 mM at 15 min in all trials then declined to approximately 1.5 mM by 30 minutes and remained at that level for the remainder of the 63% exercise task (Figure 4). Blood lactate increased to over 8 mM during the HI effort and returned almost completely to resting values at 15 minutes of recovery (Figure 4). There were no differences between **C** and **G** in blood glucose or lactate levels.

### Substrate Oxidation

**Figure 5:**
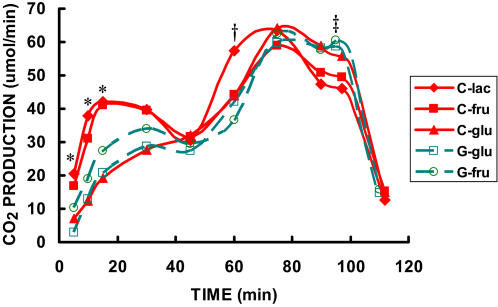
Time-course of CO_2_ production (^13^CO_2_ appearance in expired air) while exercising at 62% for 90 minutes followed by exercise to exhaustion at 86% of VO_2_max during three C trials and two G trials. The three C trials and two G trials were randomly ordered. * significantly different from G-glu (p<0.05). † significantly different from all other substrates (p<0.05). ‡ G-glucose significantly different from C-lac (p<0.05). Data are means. SE were omitted for the sake of clarity.

**Figure 6 pone-0000927-g006:**
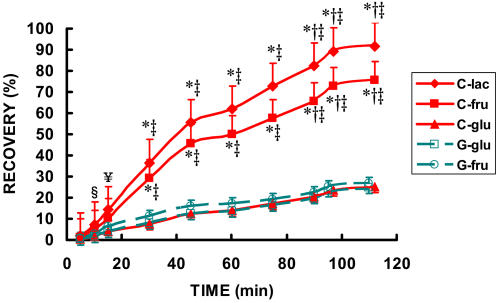
Time-course of the cumulative recovery of ^13^CO_2_ in expired air while exercising at 62% for 90 minutes followed by exercise to exhaustion at 86% of VO_2_max during three C trials and two G trials. The three C trials and two G trials were randomly ordered. § cumulative recovery of lactate different from cumulative recovery from G-glu (p<0.05). ¥ cumulative recovery lactate different from cumulative recovery from glucose from both drinks (p<0.01). * cumulative recovery different from cumulative recovery from glucose from both drinks and fructose from G (p<0.01). † cumulative recovery different from cumulative recovery from glucose and fructose from both drinks (p<0.05). ‡ time point different from all other time points (p<0.01). Data are means±SE.

There were clear differences between **C** and **G** in substrate oxidation patterns as indicated by the excretion of ^13^CO_2_ in breath, whether considered from the standpoint of tracer oxidation rate (Figure 5), or the fractional oxidation rate (Figure 6). The labeled CO_2_ production rate following **C**-lac ingestion rose in the first 10 minutes to almost 40 µmol·min^−1^ (p<0.01 v. **G**-glu) and exceeded 40 µmol·min^−1^ by 15 minutes of exercise (p<0.01 v. **G**-glu). The next most rapidly oxidized substrate was fructose from CytoMax (**C**-fru). ^13^CO_2_ production from ingested **C**-fru started at approximately 17 µmol·min^−1^ and increased rapidly also to over 40 µmol·min^−1^ at 15-min. Tracer CO_2_ production rates following ingestion of lactate and fructose labeled CytoMax appeared to decline from 30 to 45 minutes (Figure 5), but only because the tracer dose was largely eliminated by that time and the remaining tracer dose was becoming vanishingly small. ^13^CO_2_ production from ingested labeled metabolites rose again after ingestion of the second dose of isotope at 45 min of exercise. **C**-lac tracer CO_2_ production rate rose far more dramatically than that from any other substrate measured upon ingestion of the second tracer dose, doubling between 45 and 60 minutes of exercise. Lactate oxidation rate peaked and declined prior to the 75-minute time point (Figure 5); again, this result probably attributable to rapid tracer oxidation followed by a lack of availability. In contrast, labeled CO_2_ production rates from second tracer doses of all other substrates peaked at or after minute 75 (Figure 5). Between minutes 75 and 90 substrate oxidation rates declined. During the HI effort, **G**-glu tracer CO_2_ production was higher than in **C**-lac (p<0.05) most likely due to the relatively slow kinetics of glucose compared to lactate such that maximal glucose oxidation lagged behind lactate for about 30 minutes. Oxidation of all exogenous tracers declined dramatically after the cessation of exercise (Figure 5).

**Figure 7:**
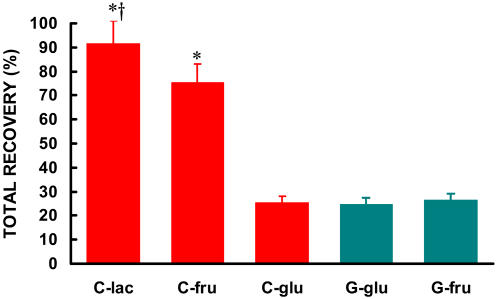
2-hour cumulative recovery of lactate, fructose and glucose from C and glucose and fructose from G during 90 minutes of steady state exercise at 62% VO_2_max followed by an 86% VO_2_max effort until volitional fatigue. Total recovery was calculated as the sum of the cumulative recovery. Data are means±SE. * significantly different from C-glu, G-glu, G-fru (p<0.01). † significantly different from C-fru (p<0.01).

The cumulative recovery rate of tracer in **C**-lac as ^13^CO_2_ was significantly larger than any other substrate in either drink (Figure 6). For the first 30 min of exercise, the fractional oxidation rate of lactate in CytoMax (**C**-lac) was 37%, or more than three times higher than from either carbohydrate source in **G** (i.e., 11% for fructose (**G**-fru) or 8% for glucose (**G**-glu) (p<0.05) (Figure 6). For **C**-lac, by minute 45, approximately 60% of the lactate had been recovered as ^13^CO_2_ in expired air, and by the end of exercise over 92% of the label given in two oral boluses of ^13^C-lactate was recovered as expired ^13^CO_2_ (Figure 7).

The only substrate that approached the relative recovery of ^13^CO_2_ from ingested lactate was fructose from CytoMax. The recovery of **C**-fru paralleled that of **C**-lac; however total recovery for **C**-fru was less at each time point amounting to 75% over 90 min (Figures 5–7).

The recoveries for glucose in CytoMax as well as both substrates (glucose and fructose) in **G** were only approximately 25% (Figures 6 & 7). The oxidative disposal of glucose was the lowest observed substrate in both beverages (**C** and **G**).

Thus, the recovery of the tracer from **C**-lac in breath was both more rapid and more complete than any of the carbohydrate sources in **G** (Figures 5–7).

### Sprint Performance

**Figure 8:**
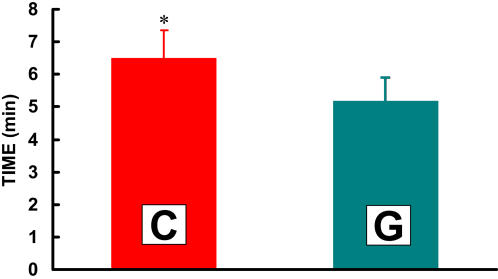
Time to exhaustion at 86% of VO_2_max after 90 minutes of cycling at 62% of VO_2_max while drinking either C or G. C is the mean±SE of three trials. G is the mean±SE of two trials. Five trials total were carried out in random order. Time to exhaustion while drinking C was significantly longer (6.5±0.8 min) than while drinking G (5.2±1.0, p = 0.05).

The cyclists in the present study rode 25% longer on **C** (6.5 min) than on **G** (5.2 min) during the high intensity (HI) portion of the exercise trial (Figure 8). VO_2_ and RER were monitored constantly during the high-intensity trials to insure maximum effort. VO_2_ peaked at 4.4 L·min^−1^ for both CytoMax and **G** during the high-intensity effort. Further, subjects exercised at 86% of VO_2_max during the HI portion of the trials while using **C** as well as **G**. RER exceeded 1.05 during all trials, so we can infer that subjects exercised maximally during the HI trials.

### Reliability Assessments

Because subjects were studied twice with **G** and three times with **C**, reliability of sprint performance could be assessed. The interclass correlation coefficient (ICC) for repeated sprint performances on subjects taking **G** was high and significant (r = 0.089, p<0.03). For repeated sprint performances when subjects were taking **C**, the ICC was again high and statistically significant (r = 0.957, p<0.04).

## Discussion

On the bases of fractional oxidation rate and cumulative recovery of tracer in expired CO_2_ collected over more than 90 min of continuous followed by high intensity exercise, results showed that lactate was used as a fuel much faster and more completely than glucose, particularly in the drink formulations tested which are typical of sports drink platforms that are commonly used. Given the plethora of scientific evidence regarding endogenous lactate oxidation in humans *in vivo*, the results in Figures 5–7 showing rapid and extensive oxidation of orally supplied lactate are not surprising [4], [5], [17], [18], [20]–[24], [26]–[41]. The rapid rate of lactate assimilation, distribution and oxidation could be predicted given its central role in linking glycolytic and oxidative metabolism, as well as the wide-spread expression of lactate-hydrogen ion symporters (referred as monocarboxylate transporters, or MCTs) in muscle and other tissues [19], [22], [23], and the presence of a sodium ion-mediated intestinal MCT [24], [25].

The initial oxidation rate of lactate was clearly superior to glucose and fructose at the outset of exercise, but equally or more impressive was the rapid assimilation and use of lactate during exercise as evident by the excretion of ^13^CO_2_ at 60 min of exercise following consumption of the tracer dose at 45 min (Figure 5). Several factors may be responsible for the more rapid rise in ^13^CO_2_ excretion following the second dose. Figure 5 exhibits classic bolus tracer kinetics. There is an initial rise in the appearance of ^13^CO_2_ from each energy substrate, most rapidly appearing from lactate, followed by a decrease in the rate of ^13^CO_2_ appearance. The decrease would be due to a diminished isotopic supply from the bolus ingested at −2 minutes. However, because the second dose of isotope was ingested prior to complete exhaustion of the first bolus of isotope, the response observed at 60 minutes is amplified due to the additional isotope to the already existing supply. In this context the high oxidation rates of lactate and other substrates during the second half of the exercise trial than at exercise onset is understandable.

The oxidation rate of fructose was intermediate between lactate and glucose, actually tracking the pattern of lactate-derived tracer excretion in breath at the beginning of exercise. The results are consistent with those of Jeukendrup and colleagues [1], [6], [7], [8], [10], [42] who showed greater tracer oxidation from glucose + fructose mixtures compared to glucose alone when >6% solutions were taken by exercising athletes. The results may indicate muscle fructose uptake and oxidation [43]. However, a more likely route of entry for fructose is that it may undergo glycolysis in the intestinal mucosa or elsewhere in the splanchnic bed, causing a rapid entry of lactate into the systemic circulation [44], [45].

As indicated in Figures 1–4, neither **C** nor **G** affected whole body metabolism or circulating metabolite levels. Hence, results affirmed that most energy (>90%) during exercise is derived from endogenous sources. However, fractional oxidation rates of exogenous substrates showed clear differences in availability for oxidation in individuals engaged in sustained intense exercise (Figures 5–7). Therefore, it is appropriate to conclude that while providing important information on overall, whole-body substrate metabolism, non-tracer derived pulmonary and blood measurements are insufficient to reveal the contributions of exogenous metabolites as utilized in the present investigation.

Results obtained in the present investigation indicate that various fuel energy substrates in sports drinks use different transport systems to gain access to the systemic circulation and cellular pathways of oxidative metabolism at different rates. Results can be interpreted to mean that the ideal sports drink should contain several different energy substrates because the absorption of any single substrate is limited by competition for its unique transporter sites. Including several substrates in a sports drink might accelerate the rate of energy absorption due to the utilization of various independent transport systems, and therefore the best way to provide energy substrate during prolonged exercise [1].

In terms of the fractional oxidation rates of exogenously supplied fuel energy substrates, the results obtained in the present investigation are likely also attributable to the presence of muscle cell (sarcolemmal) transport systems as well as the intracellular pathways of energy substrate utilization. The preference of working muscle for lactate over glucose has been established in combined lactate clamp and tracer studies [36], [37], [38]. The apparent preference for fructose over glucose is likely attributable to the conversion of fructose to lactate and subsequent use of lactate derived from exogenously supplied fructose (*vide supra*).

Lactate can be taken up by more than one type of tissue during exercise, so it is not known which tissue oxidized lactate in this study. However because working muscle accounts for most of the whole-body pulmonary oxygen consumption during exercise, it can reasonably assumed that working muscle accounted for most of the observed [U^13^C]lactate oxidation. Although it is well established that lactate is also the major gluconeogenic precursor that is taken up by the liver and converted to glucose which can be released to maintain blood glucose [46], if ingested lactate first went to the liver and was converted to glucose, an entirely different kinetic response from that observed would be expected. The appearance of ^13^CO_2_ from lactate in the breath would have been far slower than the rapid lactate fractional oxidation rates observed in the present study if gluconeogenesis was a major route of disposal of exogenously supplied lactate. Therefore, it is appropriate to conclude that the rapid oxidation of lactate in comparison to glucose is consistent with results of vascular lactate tracer and clamp procedures showing preferential and direct oxidation of lactate over glucose, and not lactate carbon converted to glucose and then oxidized. The results of the present investigation are important from another aspect because they indicate that at the exercise power output studied, splanchnic blood flow is sufficient for assimilation of the exogenous carbohydrate supply [46].

A result from the present study that is difficult to explain is the discrepancy in fructose oxidation following **C** or **G** ingestion. One possibility involves the form in which fructose is incorporated into the respective drinks. CytoMax uses crystalline fructose whereas **G** uses high-fructose corn syrup (HFCS). Perhaps the fructose in the HFCS is saturating the GLUT5 in the small intestine, thus fructose entry/uptake is transport-limited. Alternatively, some other component or characteristic of HFCS may have affected availability of fructose in G.

Prior to the present research, two other studies sought to determine the efficacy of lactate as a sports drink component [5], [16]. Neither study utilized tracers to quantify metabolite fractional oxidation rates as was done in the present investigation. Fahey et al. found that in comparison to a glucose polymer, a PolyLactate-glucose polymer combination was able to maintain blood glucose at least as well as glucose polymer alone. This is likely due to the role of lactate in gluconeogenesis. Further, Fahey et al. found that PolyLactate resulted in greater bicarbonate concentration in the blood of their subjects. This greater bicarbonate concentration could have an implication on enhanced performance during high-intensity exercise. This will be discussed in detail below.

Swensen et al. compared endurance performance of men taking one of either two glucose polymer-based sports drinks, one with PolyLactate (and less glucose polymer) and glucose polymer alone [16]. Swensen et al. concluded, however, that adding PolyLactate to a glucose polymer-based drink was not beneficial, that is, it did not enhance endurance time during steady-state exercise. Total carbohydrate was well controlled between trials such that subjects received the same amount of carbohydrate (7% solution, 0.3 g CHO/kg body wt) for each sub-maximal trial. The subjects in the Swensen study exercised at a sustainable energy expenditure in both trials (70% VO_2_max). The investigators did not conduct repeated trials with the two beverages, so reliability of exercise performance could not be assessed. Nonetheless, it is no surprise that there was no difference in time to exhaustion with steady-state exercise which may have represented an inadequate challenge to energy substrate delivery pathways to require exploitation of parallel energy substrate transport systems for optimal performance. In contrast, in the present investigation, to simulate conditions that occur in sport, the subjects were challenged with a non-steady state, unsustainable sprint-like exercise task (86% VO_2_peak to exhaustion) after a prolonged bout of exercise. This substantial challenge to energy metabolic pathways made apparent the benefit of the more diverse formulation in CytoMax, which includes PolyLactate.

Besides the well-established role of lactate as an essential intermediate between glycolytic and oxidative metabolism, the increased performance occurred could be partially explained by the enhanced buffering capacity of PolyLactate. It has been demonstrated that there is an increased bicarbonate and blood pH during exercise in subjects consuming a drink with PolyLactate as opposed to glucose polymer alone [5]. The lactate in CytoMax is able to stoichiometrically scavenge protons because the lactate anion is the salt of the acid. By scavenging protons, the lactate in **C** acts to spare bicarbonate during periods of high proton efflux from skeletal muscle or intense exercise (which may explain why five of six subjects sprinted longer after prolonged, hard continuous exercise when consuming **C** than they did when consuming **G**). As well, the disposal of exogenous lactate as lactic acid via oxidation or gluconeogenesis results in stoichiometric removal of protons (H^+^). That such a buffering, or other, effect of PolyLactate may have been in operation is suggested because the effect on sprint finishing performance (Figure 8) can not be explained on the bases of respiratory gas exchange (Figure 2) or blood metabolite levels (Figures 3 and 4). Results of the present study confirm that the energy substrate platform of sports drinks dramatically affects athletic performance.

While subjects in this study sprinted significantly longer when taking **C** vs. **G**, the number of study participants was small. There is less statistical power in a small sample size making it more difficult to reach statistical significance, thus the fact that statistical significance was reached, gives additional credence to the present findings. In this regard, it is to be reiterated that highly fit and experienced cyclists were used. The drinks (**C** and **G**) were matched in color and taste, and subjects were naïve to the beverage and tracer given on any particular day. Further, subjects were not informed of their sprint times, but a financial inducement was offered to the participant with the longest sprint time. Most importantly, excellent test reliability measures for G and C, 0.89 and 0.96, respectively, were obtained. Hence, it is reasonable to conclude the statistical difference in exercise performance was real.

In summary, this is the first report of greater fractional oxidation of lactate in comparison to other carbohydrate energy substrates during exercise. As well, the presence of fructose as an ingredient in an energy-electrolyte hydration beverage provides and advantage over glucose alone in terms of providing energy to an exercising athlete. By providing PolyLactate™ as well as fructose, glucose and glucose polymers as CHO-energy forms, CytoMax possesses clear advantages in terms of providing rapid and sustained energy allowing superior sprint finishing performance in comparison to a popular HFCS-based sports drink.

## Methods

### Body composition and maximal oxygen consumption

Six male subjects were recruited from the local cycling community. After an overnight fast, body composition was measured by air displacement densitometry (Bod Pod, LMI, Concord, CA) using the Brozek equation [47]. Maximal oxygen consumption was assessed on a Monark model 839E electrically-braked cycle ergometer (Vansbro, Sweden) using a continually increasing power output protocol. Power output started at 100 watts and was increased in 50 watt increments every two minutes until 400 watts and increased in 25 watt increments after that. All subjects reached at least 400 watts, and proceeded until volitional fatigue. Maximal oxygen consumption was identified as the greatest oxygen consumption value attained during the test. VE, VO_2_, VCO_2_, RER ( = VCO_2_,/VO_2_,) were assessed by indirect calorimetry (ParvoMedics 2400; Salt Lake City, UT).

### Exercise-sports drink trials

Subjects reported to the lab at about 0800 on five separate occasions for their submaximal exercise trials. They first completed a 24 hour dietary log. Subjects performed continuous exercise (CE) at 62% of VO_2_max for 90 minutes after which they exercised at 86% of VO_2_max (high-intensity effort, HI) until volitional fatigue. Fatigue was defined as the inability to maintain ergometer power output at a cadence above 40 rpm. Subjects then recovered for 15 min at 50% of the HI power output.

### Drinks

Subjects received isocaloric volumes (250 ml, 55 kcal) of either CytoMax (**C**) or a leading brand (**G**). Citrus Blast (CytoMax) and a citrus-flavored version of **G** were used to match color and taste and were obtained commercially. Drinks were administered two minutes prior to exercise (−2), and then every 15 minutes during exercise until the 90 minute time point. To trace the components of **C** or **G**, 100 mg of uniformly-labeled ^13^C-glucose (glu), ^13^C-fructose (fru) or ^13^C-lactate (lac) (Cambridge Isotope, Cambridge, MA) was added to the drinks consumed at −2 and 45 minutes of exercise. The total molar ^13^C-label dose was not different between the 5 trials, and the order of trials was randomized and subjects were blinded from knowledge of the drink provided on any particular day. Trials for respective subjects occurred about every 1.0 to 1.5 weeks. Subjects tolerated all drinks very well, and none reported any GI distress or other side effects from consuming the drinks as indicated in a post-trial questionnaire.

### Blood samples

Arterialized blood samples were collected from a heated superficial hand vein at rest (−2 minutes), 5, 10, 15, 30, 45, 60, 75, 90 minutes of exercise, the end of exercise (∼ min 95 & 97 for **G** and **C**, respectively) and 15 minutes after the cessation of exercise. Blood samples were immediately transferred to ice-cold 8% perchloric acid, weighed, spun, supernatants decanted and stored at −80°C until assayed for glucose and lactate.

### Assays

Glucose was analyzed using the HK-based assay that couples G6P with GPDH that is a NADH-linked assay (Pointe Scientific, Canton, MI). Samples were read at 340 nm.

Lactate was assayed enzymatically by the method of Gutmann and Wahlefeld [48].

### Isotopically-enriched CO_2_ collection

Gas samples for ^13^CO_2_ analysis were collected using a tube inserted into the mixing chamber of the metabolic cart. The tube was connected to a three-way stopcock that had a 30 cc syringe attached via the luer fitting. On the last port of the stopcock an 18 gauge needle was fitted. Two minutes prior to the sample time, air was drawn into the syringe using slow-even strokes. Two to three flushes were taken prior to every sample. After the sample was drawn into the syringe, an Exetainer™ tube was inserted onto the 18 gauge needle. Ten cc of the sample were drawn into the Exetainer™ tube. Duplicate samples were collected for each time point. Samples were shipped to Metabolic Solutions (Nashua, NH) for ^13^CO_2_ enrichment determination by IRMS. Data were reported as atom percent excess (APE). Labeled CO_2_ production rates were divided by 0.94 to correct for bicarbonate retention.

### Calculations

CO_2_ production from ingested substrate (in µmole/minute) was calculated using (VCO_2_/22.4)×(APE/100)×1,000,000 where:

VCO_2_ is CO_2_ production in L/min,

22.4 is the molar equivalent for gas volume

APE/100 is atom percent excess divided by 100 to get the required fraction

1,000,000 converts moles to µmole.

Isotope recovery was calculated by taking the average ^13^CO_2_ production rates for two successive time points and multiplying by the number of minutes in the time between the time points {recovery (µmole) = [(rate @ T1+rate @ T2)/2]×time interval}.

Percent recovery was calculated by dividing the isotope recovery by the amount of isotope consumed by the subjects (i.e. µmol ^13^C recovered/µmol ^13^C isotope consumed (FW of the lac, glu, or fru + the correction for uniformly-labeled substrate)×100. We refer to percent recovery as fractional oxidation rate as it represents the percentage of exogenous substrate consumption that was oxidized. Cumulative percent recovery was calculated by summing successive percent recoveries while accounting for previously excreted isotope.

### Statistical analysis

Two-way analysis of variance (ANOVA) using repeated-measures was used in the analysis with time and type of drink as within subject factors. When indicated by a statistically significant ANOVA test, differences were further investigated using a series of post-hoc paired comparisons with Bonferroni adjustment. For the presentation of data in figure form and statistical analysis, time to exhaustion data from two **G** trials were averaged as were the times to exhaustion from three **C** trials. A paired t-test was used to assess differences between drinks in time to exhaustion. Interclass correlation coefficients were calculated to assure consistency within the **C** and **G** trials during the HI portion of the exercise trials. Significance level was set at α = 0.05.

### Human subjects

Protocol and procedures were reviewed and approved by the California State University Chico Human Subjects in Research Clearance (HSRC) committee. Informed consent was obtained from every subject prior to admission into the study and protocols and procedures were in full compliance with the Declaration of Helsinki and all local, State and Federal laws.
